# Triticale in Italy

**DOI:** 10.3390/biology12101308

**Published:** 2023-10-04

**Authors:** Nadia Faccini, Caterina Morcia, Valeria Terzi, Fulvia Rizza, Franz-Werner Badeck

**Affiliations:** Consiglio per la Ricerca in Agricoltura e l’Analisi dell’Economia Agraria—Centro di Ricerca Genomica e Bioinformatica (CREA-GB), Via San Protaso 302, 29017 Fiorenzuola d’Arda, Italy; nadia.faccini@crea.gov.it (N.F.); caterina.morcia@crea.gov.it (C.M.); fulvia.rizza@libero.it (F.R.); franz-werner.badeck@crea.gov.it (F.-W.B.)

**Keywords:** triticale varieties, breeding, varietal recommended lists, certified seeds

## Abstract

**Simple Summary:**

Triticale is a human-created cereal, originally bred with the aim of combining the useful traits of *Triticum* (high yield potential and good grain quality) and *Secale* (vigor and resistance to diseases and abiotic stresses, including soil conditions). Triticale has several final destinations; herbages and grains are mainly used for livestock feeding, the grains can be used for niche food and the whole plant can be used as energy crop. In 2020, more than 13 million tons were harvested in Europe. Italy contributes to European production with a minority share that is equal to approximately 0.5% of the entire production. An overview of the varietal landscape, major uses and perspectives for triticale cultivation in Italy is presented.

**Abstract:**

Triticale is currently grown throughout the world with a wider diffusion in Europe, with Poland, Belarus, Germany, France and Spain as major producers. Although triticale occupies a very small fraction of the Italian cultivated land (16,000 ha of harvested area, mean value of the past 5 years), a continuous interest for this crop and its possible uses explains the work and progress made by breeding activities in different periods. The aim of this review is to report some experiences related to the cultivation of triticale in Italy. A general long-term view of the performance of triticale varieties in Italy has been distilled from a large amount of data derived from the pluri-decennial Italian national variety trials network. This activity, historically coordinated by CREA-GB, extends over several decades and examines the agronomic performance, in different Italian environments, of the most widespread and emerging varieties of triticale. Indications on new breeding targets can be deduced from the elaborations in the frame of both climatic change and market demands.

## 1. Introduction

Triticale is an amphidiploid produced by doubling the number of chromosomes of the sterile intergeneric hybrid between the female parent wheat (*Triticum* ssp.) and the male parent rye (*Secale* spp.), thereby combining the genomes A and B or A, B and D of wheats with the R of rye. The interest for this human-created cereal was stimulated by the aim to combine the useful traits of *Triticum* (high yield potential and good grain quality) and *Secale* (vigor and resistance to diseases and abiotic stresses, including soil conditions) [1]. Triticale, or better, triticales, are distinguishable by origin as primary, octoploid or hexaploid when derived from the hybridization of the rye genome with common or durum wheat, respectively, and secondary triticales are bred by crossing between primary triticales. The evolution of triticale from a botanical curiosity to a species of agricultural interest relies mainly in the development of the secondary hexaploids in the 1950s, marking the beginning of breeding programs in Europe and North America [2,3,4]. Introduced into cultivation at the beginning of the 1970s, the secondary hexaploids were characterized by a greater cytological stability and higher agronomic characteristics for the partial replacement of the chromosomes of rye with wheat. An updated 360-degree view of this cereal is given in the review by Del Pozo et al. [5].

Triticale is currently grown throughout the world with a wider diffusion in Europe, with Poland, Belarus, Germany, France and Spain as major producers. In 2020, more than 13 million tons were harvested in Europe [6]. Italy contributes to European production with a minority share that is equal to approximately 0.5% of the entire production. However, the yield is at a good level: the average yield of the past five years in Italy is, in fact, equal to 45,000 hg/ha compared to an average European yield of 39,000 hg/ha [6].

Triticale, in Europe and in Italy, is mainly used for livestock feeding. As a feed grain, it is already well established and of high economic importance [7]. The grain is particularly easy to digest and adapted to the nutrition of monogastric animals, especially in the first phases of growth, due to the starch quality and the amino acid composition; it is rich in lysine compared to maize and wheat. The use of triticale as a forage (green fodder or silage) is prevalent for the feeding of ruminants.

In some countries, grains have also been employed for food [8]. But the use of triticale for producing special breads, malt, dietetic preparations and macrobiotic food in these countries is still at the level of niche. In areas such as Eastern Poland, characterized by acidic soils and cold climates, triticale has replaced rye in the production of bread.

More recently, triticale has received attention as a potential energy crop, and research is currently being conducted on the use of the crop biomass in bioethanol production. Szempliński et al. [9] underlined that energy efficiency is one of the key priorities of a sustainable crop, and in this regard, the energy efficiency ratio of winter triticale has been found to be remarkably high at all levels of agricultural inputs.

The breeding effort in triticale started from the hypothesis to combine the favorable traits of wheat and rye, and as expected, this resulted in an ample genetic variability derived from the combination of two species [10]. The original hypothesis that the useful traits of two species can be combined in a superior culture can be reconsidered now, in the light of the results of decades of selection and cultivation, as reviewed by Arseniuk [11]. Particularly informative for this purpose are the Country Reports on triticale adoption in different worldwide cultivation areas collected in Mergoum and Gómez-Macpherson [3]. Information on triticale in Italy is missing in this document. The aim of this review is therefore to contribute to fill this gap by focusing on the experience in Italy, a Mediterranean country with remarkably diverse environments and agricultural systems, where an increased interest for triticale, which was traditionally cultivated in a limited area, has been registered in recent years.

Here, we present a qualitative review of the literature on the cultivation and use of triticale in Italy, along with publicly available statistical information.

## 2. Triticale Varieties in Italy: Historical Notes and Current Italian Varietal Landscape

The interest in triticale as a crop in Italy dates back to the early 1970s when the first breeding program, developed using CIMMYT (Centro Internacional de Mejoramiento de Maiz y Trigo) segregating populations, was aimed at breeding cultivars adapted to Italian environments. The first Italian cultivar, “Mizar”, released in 1980 by ENEA (Roma), featured a spring habit, a medium plant height and good resistance to lodging and showed a high yield potential and good adaptability to less fertile soils [12]. It was followed by “Rigel”, a tall cultivar bred from a cross between a spring habit and a winter habit line and adaptable to various national environments. The varietal landscape significantly increased in the 1990s with the development of breeding programs in both private and public sectors. Genetic variability enclosed in the segregating populations bred at CYMMIT was exploited in combination with the cultivated germplasm of European (mainly winter types from Poland and France) and Australian origin. In the 1990s, twelve new cultivars were released and added to the Italian National Varietal Registry.

From the 1990s to today, new genotypes of high agronomic value with differentiated morpho-physiological traits for a better adaptation to different cultivation environments were introduced. A number of cultivars were made available to growers, mainly with an alternative or winter growth habitus, variable lengths, dimensions and lodging tolerances, with heading dates ranging from a contemporary period to early varieties of barley until dates posterior to the later wheat. The varietal median heading dates in national yield trials range from mid-April to the end of May in Northern Italy and from early April to mid-May in Southern Italy.

**Figure 1:**
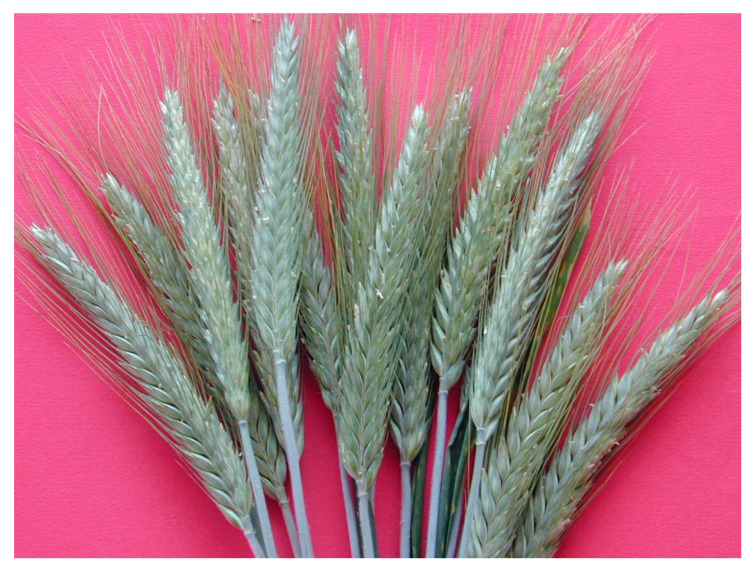
Spikes of the Oceania cultivar.

Among such varieties, a turning point is represented by the introduction of the cultivar, “Oceania” (Figure 1), which was released in 2002 by the formerly Istituto Sperimentale per la Cerealicoltura (now CREA-GB), a rare example of a cultivar with high yield potential and excellent agronomic stability, thanks to its adaptability to different types of stress. The early heading date and the low stature of this cultivar have been proven as useful traits, allowing for the crop to escape drought stress in areas subjected to water scarcity. Due to its productive performance in different environments, “Oceania” remained for many years in the list of recommendation for the cultivation areas of Northern, Central and Southern Italy, resulting from the annual national trials network, and is still competitive with the most recent cultivars, especially in the south. Recently, the spring variety, Oceania, was evaluated in a mixture with the intermediate Bienvenue in the typical Mediterranean environment of Sardinia [13], and it was found that its performance as a herbage of the mixture was improved in comparison with single varieties, and that even the grain yield stability was increased, probably due to the greater resilience of the mixture to abiotic stresses.

Although triticale occupies a very small fraction of the Italian cultivated land (16,000 ha of harvested area, mean value of the past 5 years) [6], a continuous interest for this crop and its possible uses explains the work and progress made by breeding activities in different periods. Now, 52 varieties, mainly obtained from Italian and European breeding programs, are included in the Italian National Register [14].

**Figure 2:**
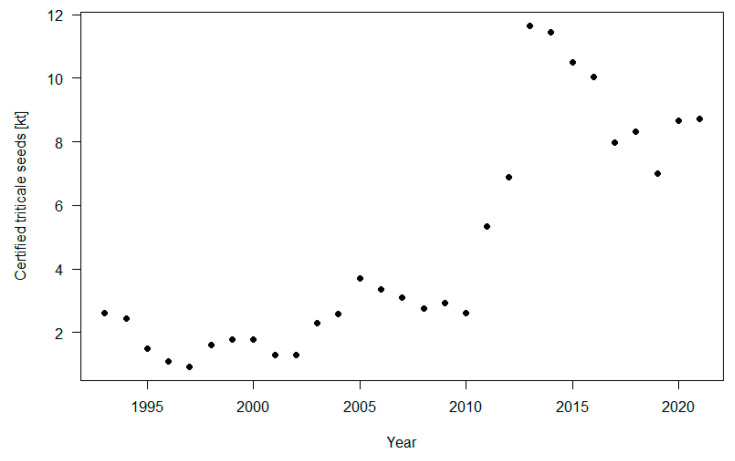
Production of certified triticale seeds in Italy (kt). The data are provided by CREA-DC (Difesa e Certificazione), formerly ENSE, Italy [15].

The interest in varietal innovation had a decisive impact on the volumes of certified seeds that are commercialized. It is noteworthy that, in the past decade, the amount of certified seeds has changed dramatically (Figure 2). In the period of 1993–2010, the certified seeds volumes were below 4 kt per year. Later, the quantities increased considerably, with values included between 5 and 12 kt. In view of the jump in the production of certified seeds in the second decade of the 21st century, it is reasonable to assume that the major increase in triticale production occurred in parallel. Focusing on the individual varieties, Table 1 reports, for the years of 2020, 2021 and 2022, the twenty major varieties for annual certified seed production. In greater detail, the percentages of certified seeds that were registered for each variety out of the total certified seeds produced are reported. Vivacio ranked first in the three years considered. A varietal turnover can be observed, and the varieties derived from Italian breeding programs are represented.

To support farmers’ decisions for the next sowing, a list of recommended varieties has been annually drawn up, in the past 30 years, by CREA-GB, as a public research and experimentation body at the service of the Italian Ministry of Agriculture. This activity, historically coordinated by CREA-GB in collaboration with several other regional institutions, examines the agronomic performance, in different Italian environments, of the most widespread and emerging varieties of triticale. The final information is structured in three lists of recommended varieties for the three areas of Northern, Central and South Italy and the islands that are made publicly available through the national technical press [16].

## 3. Triticale Production in Italy and Destination of Use

The statistical data on the triticale production and yield in Italy are only available for a short period in the late 1980s of the 20th century and then from 2016 onwards. In the intermediate period, lumped figures for triticale and emmer were published. 

The average annual production in the late 1980s amounted to ca. 14 kt, while it was 68 kt on average in recent years [17,18]. The higher production in recent years compared to the late 1980s was due to an increase in the average yield from 3.6 to 4.5 t ha^−1^ and a substantial ca. 4-fold increase in the area cultivated with triticale.

**Figure 3:**
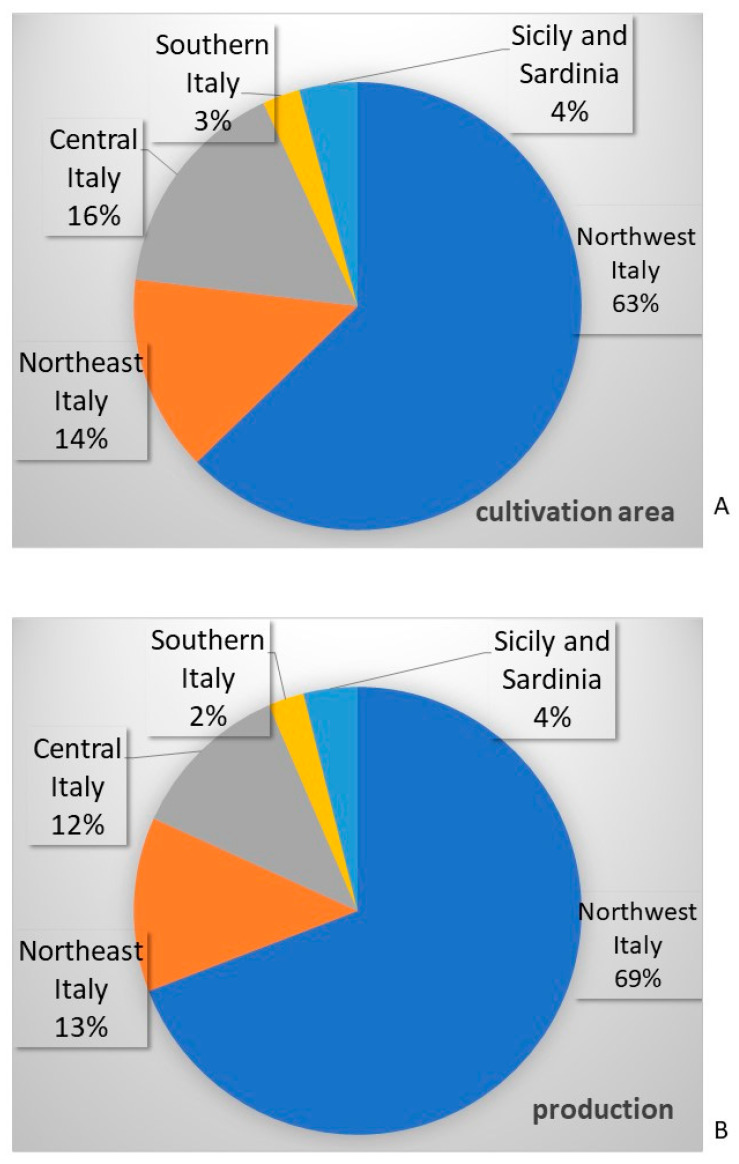
Cultivation areas (**A**) and production (**B**) in the five macro areas of Northwest Italy, Northeast Italy, Central Italy, Sothern Italy, Sicily and Sardinia. Mean values of the past three years are reported [18].

In the past three years, the overall Italian triticale production was of 66 kt, with a cultivation area of 14,550 ha [18]. Figure 3 shows the productions and the percentages of cultivated areas in the five Italian macro-areas of the northwest, northeast, center and south, including the major islands (Sicily and Sardinia). The regions of Northwest Italy hold the highest share in triticale production, particularly Piemonte and Lombardia, followed by the northeastern regions (Veneto, Friuli-Venezia Giulia and Emilia-Romagna) and Central Italy (Toscana, Umbria, Marche and Lazio). Sicily and Sardinia contribute with a mean annual production of 25 kt, whereas the southern regions of Molise and Calabria produce around 16 kt.

Comparing the production levels and cultivation areas, triticale ranks fifth among small grain cereals cultivated in Italy, after durum wheat, bread wheat, barley and oats. Its production values and cultivation area are much lower than those of wheats, but significantly higher than those of rye. Table 2 shows the mean values (years of 2021, 2022 and 2023) of the production and cultivation area of the major small grain cereals cultivated in Italy.

In Italy, triticale is now mainly used as feed; the whole plant used as fresh or silage feed at heading or milky-wax maturity has in fact proven to be viable in terms of both quantity and quality, being comparable in energy value to other winter-sown cereals. 

Work is in progress to study genetic variability for specific quality traits that are useful for feed, taking into account the frequent events of water scarcity that are affecting more and more regions of Northern Italy. An increased interest in the use of winter-sown triticale as a substitution of maize to produce typical swine and beef high-added-value products has been registered because of the lower fertilization and water demands of triticale relative to maize.

An interesting observation regarding this point was made by Cosentino et al. [19] in milking cows. The impact of a corn-silage-based diet was compared with that of a triticale-silage-based diet. No significant differences in the production level or milk composition were observed between the two diets, as they were characterized by the same energy and protein contents. However, a significant difference was observed in the water footprint: the largest water footprint for animal production comes from the feed they consume, and a triticale-based diet saves about 15% of water in comparison with a corn-based diet. The adoption of triticale silage is therefore important to formulate low-water-footprint diets.

Noya et al. [20] completed a comparative life cycle assessment of the three major feed cereals (i.e., wheat, maize and triticale) cultivated in the Po Valley (Northern Italy), the most important agricultural area in Italy for livestock farms and agro-industries. After a global environmental analysis, triticale was found to be a valid alternative to maize, thanks to its lower requirements in intensive agricultural practices and mineral fertilizers and thanks to the fact that no irrigation in the triticale cropping system is required. 

Giuberti et al. [21] showed that the starch digestion potential and pGI are extremely variable among cereals that are commonly utilized in pig nutrition, including triticale, that has the peculiarity of a high protein content.

Bioenergy production is the other main destination of use for triticale in Italy. Triticale grain has been employed with good results for the production of bioenergy (biofuels) and the cultivation system has been studied for optimization with this aim. The unfavorable ratio between grain and straw can promote the use of significant amounts of straw of high quality in the field of bioenergy without encountering ethical problems, not competing with the use as “feed” (limiting) and “food”.

In response to this requirement, the Biogasdoneright™ model, based on the double-cropping system along with digestate fertilization and minimum tillage, was proposed. Triticale varieties were evaluated in such a bioenergy production system, and it was found that it is a suitable energy crop for biomethane production [22].

In recent years, the use of the whole plant as a source of renewable energy has attracted high interest. Some results have already been achieved, showing the high yield of biogas that makes triticale a good choice for biomass production [7,9,23,24,25,26]. Further studies are necessary to identify a mix of sustainable products to valorize the use of crop residuals and further minimize the competition with the production for human and animal nutrition.

Up to now, the use of triticale for food has been marginal in Italy. The suitability of triticale flour for breadmaking, in comparison with wheat, is limited by the lower gluten quantity and quality, the lower falling number, dough stability and development time. What is more interesting is its use for unleavened baked products, such as biscuits and crackers. Recently, Piazza et al. [27] formulated triticale-based biscuits with increasing substitution levels of malted triticale flour. The technological and nutritional characteristics of the cookies obtained indicated that the substitution of triticale flour with triticale malted flour can increase the total dietary fiber and ash contents. Increasing the level of malted flour, however, increases even the reducing sugars and the in vitro-predicted glycemic index (pGI). The technological properties of triticale flour, both native and malted, suggested that this cereal can be used to formulate biscuits with high dietary fiber.

In conclusion, at present, there is an increased request in Italy for new genotypes for use as feed and for renewable energy production. New needs for healthy feed production, the spread of *Diabrotica* in the maize cultivations of the Po Valley and the presence of mycotoxins in corn production have emerged, making triticale an interesting crop alternative. The modern breeding programs take into account these recent needs by focusing on improving the biomass yield and quality and selecting genotypes with a high productivity of the whole plant and resistance to environmental stresses. Detailed knowledge of the factors affecting the biomass quality for animal nutrition on the one hand and for bioenergy production on the other hand is now strategic. Mechanisms involving components that interact with the defense mechanisms protecting the plant from attack by enzymes produced by pathogens and the stability of the plant need to be considered. Useful traits for biomass productions are the maximum production of digestible organic matter (fiber + starch), a high biomass yield, the ability of the plant to stay green at harvest, resistance to fungi and mycotoxin contamination of the plant, and stem properties.

Important metabolic changes (such as changes in the levels of cellulose/lignin) must be pursued without limiting the productive potential of the plant, keeping the useful characters selected in the course of time such as the levels of defense against pathogens and the resistance to lodging.

For such breeding objectives, several genetic maps have been developed, and quantitative trait loci and candidate genes linked to important triticale traits have been identified, as reviewed by Golebiowska-Paluch and Dyda [28]. Valuable QTL regions have been identified after multi-locations and pluriannual trials, finding that some regions on the rye chromosomes, 4R, 5R and 6R, play key roles in the resistance to biotic and abiotic stresses.

## 4. Triticale Breeding in Italy: Yield as the Trait of Main Interest

Starting from the observation that feed is the primary destination and that the quality traits of triticale were not recognized by the market quotations, an increased yield capacity is the trait of major interest. Increasing the grain output and biomass for forage (given that the milk forage unit per kg of dry matter is positively correlated to the grain yield) is therefore essential to enhance the triticale acreage in the Italian agricultural reality. However, it is well known that yield is a complex trait, and it is positively correlated with all of its components, such as the number of ears and their length, the number of fertile spikelets, the number of grains and the thousand grain weight, among others. All of these components are influenced by environmental and agronomic factors and are strongly determined by genotypic factors, but also by GXE interactions and the compensation between the yield components [29].

At the beginning of this millennium, the study of the varietal performance in various national environments, including Italian ones, made it possible to identify those traits, which can be enhanced to potentially enable higher yields.

However, a warning on this point was raised by Kozak et al. [29]. These authors found that in genotypes with a similar average grain yield, the yield components can contribute to the final value in different ways, and therefore, it is not trivial to give any general advice on which component of the triticale grain yield is the most important.

Focusing on the triticale biomass production level in Italy, when the whole plants are harvested at heading, Delogu et al. [30] showed that triticale can produce 9–10 t ha^−1^ of dry matter in a fertile environment, like the Po Valley in Northern Italy, and can produce 6–7 t ha^−1^ in typical Mediterranean environments, like Sardinia. At milky-wax maturity, the yield reaches 18–20 t ha^−1^ and 11–13 t ha^−1^ of dry matter in the two cases, respectively.

**Figure 4:**
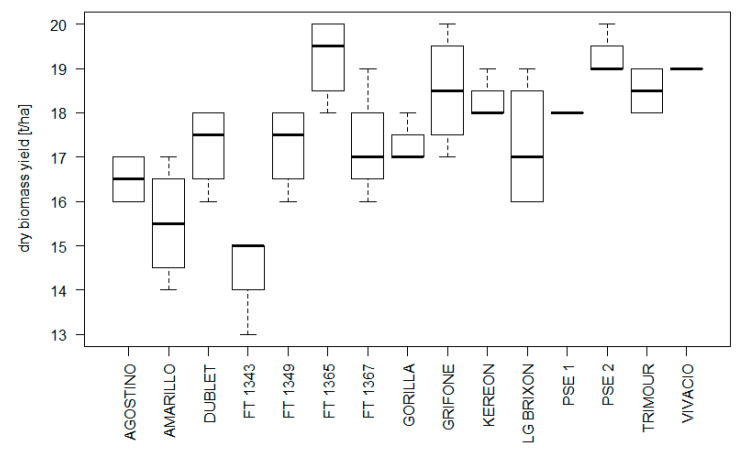
Dry biomass yield values at milky-wax maturity in triticale varieties and advanced breeding lines evaluated in Fiorenzuola d’Arda experimental field. The experiment was performed using a completely randomized block design, with 4 replicated blocks applying standard local agronomic practices.

However, it is noteworthy that a wide variability exists among different triticale varieties for a milky-wax maturity yield (Figure 4). A biomass test was conducted in Fiorenzuola d’Arda in 2016, evaluating a total of 15 genotypes including both registered varieties and advanced breeding lines. The whole plant was harvested at the milky ripening stage (almost waxy) equivalent to an average dry matter content of 29%, which is reached 15–20 days after the heading date. On average, the trial produced 60.4 t/ha of green biomass and 17.6 t/ha of dry matter, with about 5 t/ha of difference between the varieties. The dry biomass production range was 19.3 t/ha–14.4 t/ha.

Triticale cultivars are therefore competitive with the major small-grain cereals and produce more biomass than wheat in Mediterranean, dry environments. It has been suggested that such better performance in the biomass increases at anthesis and at maturity is mainly due to the greater RUE (Radiation Use Efficiency) of triticale in comparison with wheat [31,32]. The lower coefficient of light attenuation has been hypothesized to allow for a better distribution of light through the canopy [32], and such a characteristic can explain the higher biomass of triticale varieties cultivated in Mediterranean environments in comparison to wheat.

Focusing on the grain yield level in Italy, the data obtained with the national varietal trials (publicly available in [16]) provide information on the regional differences and time trends.

**Figure 5:**
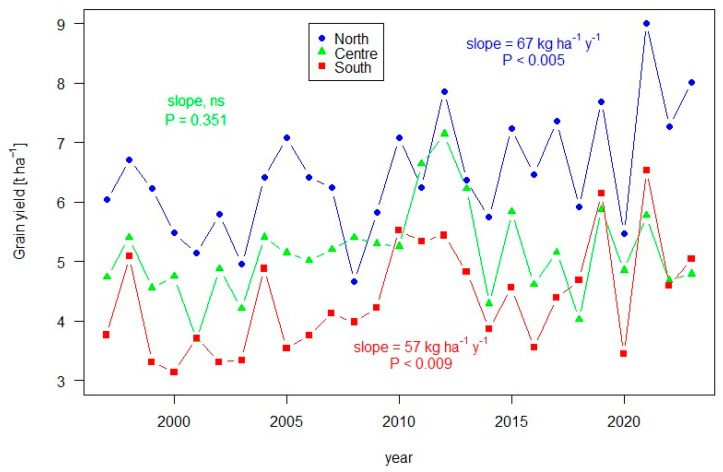
Triticale grain yield in the northern, central and southern test sites of the Italian national yield trials. The curves show yield averaged across cultivars and sites within the three geographic zones. The results of the trials are published with the annual Special Issue on minor cereals in the journal *Informatore Agrario* in the Italian language.

Figure 5 summarizes the average grain yield recorded in the period of 1998–2023 at trial sites in the three Italian macro-areas. Each data point is the mean value of the yield obtained from a panel of varieties cultivated in different environments located in Northern, Central and Southern Italy, plus major islands (Sicily and Sardinia). Significant increases in the grain yield have been registered along this temporal window in both Northern and Southern Italy. According to the reports on the national trials [16], the factors limiting production that were reported as more recurrent were both abiotic stresses, such as drought and high temperatures at the time of grain filling, and biotic stresses. Among the plant diseases, rusts have had the most negative impact in many years, especially in the Central Italian fields. 

Previous studies underlined that a peculiar feature of triticale is the constitutively high number of fertile florets per spike [32,33] in comparison with other small-grain cereals. This trait has been suggested to be a key component of production in a climate change scenario; the grains per unit area affect the shape variations in the grain yield under different levels of water availability [32,34].

Motzo et al. [34] highlighted that the yield performance of a variety is dependent on the environmental conditions in the various phases of the cultivar growing season. A longer pre-anthesis period can have a positive impact on the yield and grains per unit area if the environmental conditions are favorable to the plant development and growth. On the contrary, if environmental stressors occur during the summer, early flowering dates can be advantageous. This point has been underlined by the impressive number of studies on triticale that were carried out with reference to the typical Mediterranean climate, characterized by moderate drought stress during anthesis and an increasing water deficit during grain filling. The field experiments carried out by Giunta et al. [35] with durum wheat and triticale under different moisture levels showed that triticale has a higher level of drought resistance, thanks to the greater capacity of roots to intercept soil water at an earlier time. Moreover, the evaluation of 271 triticale lines in the Mediterranean environment of Sardinia demonstrated that large kernel numbers (m^−2^) are essential to obtain high grain yields [36]. The authors concluded that “*This highlights the importance of the pre-anthesis period in achieving high yields, even in conditions of increasing drought stress during spring, not only for its effect on the sink formation (kernels m^−2^), but also because it affects the source capacity allowing high rates of grain filling in the post-anthesis period*”.

In consideration of climate changes, a prudent breeding strategy may be to operate a divergent selection, both for late and early flowering, to ensure greater resilience to environmental changes.

**Figure 6:**
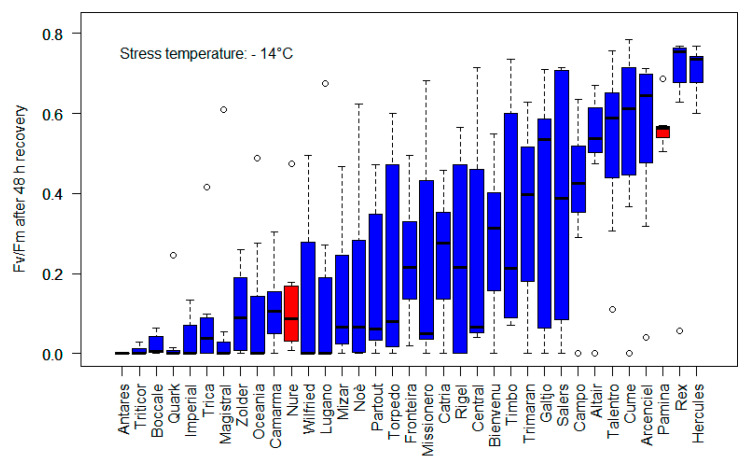
F_v_/F_m_, indicator of freezing damage to PSII, measured after freezing stress at −14 °C and 48 h of recovery under acclimation conditions. The values reported are the means of seven biological replicates. Red bars stand for two barley varieties used as references. A ratio of 0.7 to 0.8 is the optimal value measured in fully recovered leaves, with lower values indicating plant stress.

This means another class of traits must be considered, such as frost tolerance, vernalization requirement and sensitivity to the photoperiod, that is determinant of the ecological adaptation of a cultivar and the agronomic success in most geographic areas. The winter hardiness (WH) and flowering time largely depend on these traits. The frost tolerance, a component of WH, is an essential trait that allows for the high-yielding cultivars to express their potential yield. While many octoploid triticale genotypes had an excellent WH a priori, mainly due to the rye germplasm, the hexaploid triticale required much breeding effort to achieve an adequate WH. During the selection process, the multiple crosses also using spring rye and spring wheats and the introduction of spring triticales in many environments have often generated cultivars that have lost the initial advantage acquired from the rye parent. Therefore, exploring the genetic diversity for WH and the developmental traits that determine the crop adaptation should have high priority. It was shown that the phenotyping for this trait can take the advantage of a laboratory test for frost tolerance evaluation [37]. Using such an approach, a wide variability was found for this adaptative trait inside a panel of triticale varieties involved in the Italian national field trials (Figure 6). The Antares and Triticor varieties were significantly less frost-tolerant than the moderately tolerant barley winter variety, Nure [37]. The frost tolerance of the varieties from Altair through Hercules (left to right) did not significantly differ from the highly tolerant facultative barley variety, Pamina [30]. While the panel studied here comprises cultivars damaged by exposure to −14 °C, the span of cold tolerance in triticale is wide, with most tolerant cultivars surviving down to −21 and −23 °C [31].

## 5. Agronomy and Forecasting for Increasing Yield

Among agronomic factors that can contribute to the yield, fertilization is a key one; nitrogen fertilization has a direct effect on the plant uptake and, consequently, on the protein in animal feed. Cazzato et al. [38] found that when triticale was grown under Mediterranean conditions, the mode of utilization and N fertilization resulted in a greater forage yield and quality, while mycorrhizal fungus inoculation positively influenced the forage nutritional quality parameters. Moreover, Caruso et al. [39] found that a good level of biomass production in Northern Italian environments can be reached even with a digestate-based fertilization; despite a lower biomass production than that obtained with chemical fertilizers, such agronomic practice can reduce environmental and economic costs.

A further point of interest for triticale sustainability has been individuated by Lestingi et al. [40]: in Italian environments, a reduced tillage intensity improved the crude protein content and digestibility of triticale grain, and a reduction in the fertilization rate (50% of the recommended) still resulted in good grain quality parameters and yields.

Similar results have been obtained in other Mediterranean environments. Salama et al. [41] reported that *Azotobacter chroococcum* seed inoculation allowed for the reduction in the mineral nitrogen rate to 50% without sacrificing the forage and grain yields. These authors underlined that in Mediterranean countries, a triticale dual-purpose production system was profitable due to the competitive prices of triticale green forage in the region.

A further point of economic relevance for triticale cultivation is the opportunity to include this cereal in a double-cropping system; in Italian environments, triticale can be harvested around the middle of May, allowing for the sowing of a next crop. In Northern Italy, double cropping often occurs as triticale silage, followed by soybean or corn. An interesting innovation was developed by a consortium of 600 Italian farmers organized as the Italian Biogas Consortium; the two harvests are ensiled and later fed to the digester to produce biogas [42]. Negri et al. [23] compared some single and double-cropping systems in terms of biogas and biomethane production per hectare. The trials were realized in farms placed in the Po Valley, Northern Italy. A better performance was registered for the double-cropping system with triticale and the maize FAO group 500.

A further interesting study supporting the economic value of triticale as carried out by Severini et al. [43]. The study was focused on the evaluation of the economic sustainability and riskiness of cover crop mulches for the organic cultivation of corn and soybean in Northern Italy. The better choice was identified in the organic soybean cultivation, coupled with triticale as a cover crop.

In addition to the list of recommended varieties, another useful support for triticale cultivation comes from the availability of a forecasting system, as reported by Bassu et al. [44]. The APSIM-Triticale model was developed by building on those developed in wheat and using the Antares and Rigel cultivars as reference varieties, which have several different traits including plant height and biomass production. The model was found to be efficient in reproducing phenology, biomass, grain yields and soil water dynamics in Mediterranean-type growing conditions. The model was exploited even to explore the best management options across the Mediterranean basin. The simulation analysis indicated that the highest yields were achieved with an early sowing date and sowing densities between 100 and 300 plants/m^2^ in the high rainfall regions for both short and tall cultivars. In particular, the adoption of short cultivars with a reduced radiation use efficiency but elevated early vigor can potentially increase the current triticale yields across different regions, seasons and management options in the Mediterranean climate [45].

## 6. Conclusions

From the experience gained in Italy on triticale cultivation, it is agreed with McGoverin et al. [46] that “*triticale could satisfy many of the hopes originally placed upon it*”.

The varietal traits that are best suited for production under changed climatic conditions and targets for further selection need to be identified in relation to the following:

The projected climate change trends indicating, especially for Southern Europe, potentially increasing limitations of agricultural production by drought and high temperature stresses. The use of winter-grown, cold-resistant small-grain cereals [47], yielding significantly more than spring ones, provides the opportunity for rain-fed production, thus reducing limitations by scarce water resources in comparison with irrigated crops.

The exploitation of the superior crop sustainability and the adaptability of triticale for cultivation in marginal areas subjected to risks that are destinated to increase: waterlogging, extreme temperature and water scarcity. The possible expansion of winter crops to higher latitudes or elevation with global warming requires sufficient frost tolerance capacity to cope with the extreme climatic fluctuations, whose frequency has already increased in recent years.

How can one further explore and exploit the wide genetic variability existing in Europe and in the world for the (unique) cultivated germplasm generated by using rye, bread wheat and durum wheat as original parents in different environments and for different purposes? A deep characterization of the existing materials optimized for adaptation and yield performance is proposed as a necessary pre-breeding step for the identification of plant ideotypes that are able to optimize the production for different destinations and for cultivation in different European regions, according to the present and future environments and market demands.

## Figures and Tables

**Table 1:** Starting from the public data reported in [15], the percentage of certified seeds of individual varieties out of the total certified seeds produced annually was calculated. The data for the three years, 2020, 2021 and 2022, and the top twenty varieties in the ranking are reported.

2020		2021		2022	
Variety	%	Variety	%	Variety	%
Vivacio	7	Vivacio	9.1	Vivacio	9.6
Tarzan	6.7	Tricanto	7.3	Tricanto	6.9
Dublet	5.8	Rigel	4.8	LG Brixon	5.8
Oxygen	5	Bienvenue	4.6	Rivolt	5.3
Cosinus	4.9	Rivolt	4.6	Alessandro SN	5.3
Bienvenue	4.5	Agostino	3.9	Genux	4.9
Tricanto	4.2	Trivalan	3.8	Trivalan	4.4
Claudius	3.7	Froome	3.7	Bienvenue	4.3
Rigel	3.4	Claudius	3.6	Brehat	3.5
Massimo	3	LG Brixon	3.4	Froome	3.2
Alessandro SN	2.9	Quirinale	3.1	Jokari	2.7
Genux	2.8	Dublet	2.9	Ruglatt	2.7
Quirinale	2.7	Alessandro SN	2.8	Satiro	2.7
LG Brixon	2.5	Oxygen	2.6	Gorilla	2.6
Alambic	2.4	Ruglatt	2.3	Flash	2.3
Forricale	2.4	Catria	2.2	Trismart	2.1
Froome	2.4	Trastevere	2.1	Agostino	2.1
Rivolt	2.3	KWS Fido	2.1	Quirinale	2
Amarillo 105	2.2	Cosinus	2	Catria	2
KWS Fido	2.1	Tribeca	2	Cosinus	1.9

**Table 2:** Production (tons) and cultivation area (ha) of the major small grain cereals cultivated in Italy. The values are the mean of three years of data (2021, 2022 and 2023) and were extracted from a publicly available database [18].

Cereal	Production (t)	Cultivation Area (ha)
Bread wheat	2,974,200	545,000
Durum wheat	3,894,600	1,251,000
Barley	1,147,900	272,000
Oat	23,000	101,000
Triticale	65,800	14,550
Rye	11,100	3400

## Data Availability

The data are contained within the article.
